# Toi Même, a Mobile Health Platform for Measuring Bipolar Illness Activity: Protocol for a Feasibility Study

**DOI:** 10.2196/18818

**Published:** 2020-08-18

**Authors:** Aroldo A Dargél, Elise Mosconi, Marc Masson, Marion Plaze, Fabien Taieb, Cassandra Von Platen, Tan Phuc Buivan, Guillaume Pouleriguen, Marie Sanchez, Stéphane Fournier, Pierre-Marie Lledo, Chantal Henry

**Affiliations:** 1 Perception and Memory Unit, Neuroscience Department, Pasteur Institute Paris France; 2 Unité Mixte de Recherche 3571, Centre National de la Recherche Scientifique (CNRS) Paris France; 3 Centre Thérapeutique de Jour (CTPJ) Troubles Bipolaires, Clinique Bellevue Meudon France; 4 Clinique du Château de Garches Garches France; 5 Department of Psychiatry, Service Hospitalo-Universitaire, GHU Paris Psychiatrie & Neuroscience Paris France; 6 Centre of Translational Research, Institut Pasteur Paris France; 7 Department of Information Systems, Institut Pasteur Paris France

**Keywords:** bipolar disorder, digital phenotyping, smartphone app, ecological momentary assessment, mHealth, mood instability, cognitive speed, affective response, big data, machine learning

## Abstract

**Background:**

The diagnosis and management of bipolar disorder are limited by the absence of available biomarkers. Patients with bipolar disorder frequently present with mood instability even during remission, which is likely associated with the risk of relapse, impaired functioning, and suicidal behavior, indicating that the illness is active.

**Objective:**

This research protocol aimed to investigate the correlations between clinically rated mood symptoms and mood/behavioral data automatically collected using the *Toi Même* app in patients with bipolar disorder presenting with different mood episodes. This study also aimed to assess the feasibility of this app for self-monitoring subjective and objective mood/behavior parameters in those patients.

**Methods:**

This open-label, nonrandomized trial will enroll 93 (31 depressive, 31 euthymic, and 31 hypomanic) adults diagnosed with bipolar disorder type I/II (Diagnostic and Statistical Manual of Mental Disorders, 5th edition criteria) and owning an iPhone. Clinical evaluations will be performed by psychiatrists at the baseline and after 2 weeks, 1 month, 2 months, and 3 months during the follow-up. Rather than only accessing the daily mood symptoms, the *Toi Même* app also integrates ecological momentary assessments through 2 gamified tests to assess cognition speed (QU*i*CKBRAIN) and affective responses (PLAY*i*MOTIONS) in real-life contexts, continuously measures daily motor activities (eg, number of steps, distance) using the smartphone’s motion sensors, and performs a comprehensive weekly assessment.

**Results:**

Recruitment began in April 2018 and the completion of the study is estimated to be in December 2021. As of April 2019, 25 participants were enrolled in the study. The first results are expected to be submitted for publication in 2020. This project has been funded by the Perception and Memory Unit of the Pasteur Institute (Paris) and it has received the final ethical/research approvals in April 2018 (ID-RCB: 2017-A02450-53).

**Conclusions:**

Our results will add to the evidence of exploring other alternatives toward a more integrated approach in the management of bipolar disorder, including digital phenotyping, to develop an ethical and clinically meaningful framework for investigating, diagnosing, and treating individuals at risk of developing bipolar disorder or currently experiencing bipolar disorder. Further prospective studies on the validity of automatically generated smartphone data are needed for better understanding the longitudinal pattern of mood instability in bipolar disorder as well as to establish the reliability, efficacy, and cost-effectiveness of such an app intervention for patients with bipolar disorder.

**Trial Registration:**

ClinicalTrials.gov NCT03508427; https://clinicaltrials.gov/ct2/show/NCT03508427

**International Registered Report Identifier (IRRID):**

DERR1-10.2196/18818

## Introduction

### Background

In clinical practice, the diagnosis and management of mood/behavioral symptoms in bipolar disorder rely on subjective information and clinician’s evaluations, thereby raising issues, including patient recall bias, decreased illness insight during acute affective episodes, and differences in clinical assessment experience [1]. In patients with bipolar disorder, mood changes are often accompanied by shifts in other behavioral patterns such as motor activity, energy, sleep, and cognitive functions [2]. In addition, many patients with bipolar disorder experience significant daily or weekly mood swings, which do not fulfill the criteria of an acute episode but are above the levels of mood/behavioral changes experienced by nonpsychiatrically ill individuals [3,4].

Mood instability in bipolar disorder increases the risk for relapse [5] and impairs daily functioning over time [6-8], indicating that the illness is still active. Evidence has shown that patients with bipolar disorder in remission who presented with emotional hyper-reactivity, which was assessed as a proxy of mood instability by using an analog self-rated scale, had significantly increased risk for cardiometabolic dysfunction and poor cognitive functioning [9,10]. A recent study using a smartphone-based mood self-monitoring in patients with bipolar disorder has shown that mood instability was associated with significantly increased perceived stress, decreased quality of life, and impaired functioning, although most of these patients were in remission during the 9-month study period [11]. These findings highlight that mood instability could provide unique additional variance in predicting bipolar illness activity. However, the longitudinal pattern of mood instability is poorly understood as it is difficult to assess validly [12,13]. Therefore, the ability to assess mood/behavior changes continuously in real time and in more ecological conditions may be an opportunity for better understanding the clinical progression of bipolar disorder and for monitoring individual treatment outcomes.

### Digital Phenotyping in Bipolar Disorder

Currently, more than 35% of the world’s adult population owns and uses a smartphone [14]. Smartphones offer the opportunity to collect a vast amount of objective, fine-grained information (eg, data on phone usage, voice features, GPS data) in real time, which may reflect behavioral patterns, thereby providing novel insights into physical and mental illnesses [15-17]. As stated by the World Health Organization, “the use of mobile and wireless technologies to support the achievement of health objectives (mobile health [mHealth]) has the potential to transform health service delivery across the globe” [18]. Different mHealth interventions have been developed and used for various medical conditions such as diabetes, cardiovascular disease, asthma, and headache [19]. In psychiatry, mHealth systems for depression, anxiety, eating disorders, schizophrenia, and bipolar disorder have been gaining traction [20-26].

Several studies have evaluated the possibility of providing remote mood monitoring for patients with bipolar disorder by using diverse digital technologies [27-37]. For example, the ChronoRecord study included daily mood charting by using a computer [36,37]. In the AMoSS (Automated Monitoring of Symptoms Severity) study, participants monitored their moods daily by using a study-specific smartphone app, and they completed their weekly mood measures by using the True Colours system [38] and by wearing movement-sensing devices to monitor multiple physiological parameters [37]. This study has demonstrated that mood and activity monitoring were well accepted and tolerated by the participants who also reported that mood monitoring assisted them in the early recognition of their mood states [34]. The MONARCA (MONitoring, treAtment and pRediCtion of bipolAr Disorder Episodes) studies showed correlations between the severity of depressive and manic symptoms self-reported by patients with bipolar disorder who were using an electronic device and the clinically rated symptoms measured using standard mood rating scales [16]. The OpenSIMPLe feasibility study, which used a smartphone-based psychoeducation program for bipolar disorder, reported high percentages of perceived helpfulness, well-being, and general health among all the participants [33]. Moodswings, an internet-based self-help program for bipolar disorder, which includes psychoeducational material and cognitive behavioral therapy elements, has reported significant reductions in mood symptoms and improvements in the quality of life and medication adherence in patients with bipolar disorder who were using this platform [27].

Despite the fast growing development of digital technologies and the excessive hype for their use in psychiatry, robust evidences in this emerging field are lacking. For example, compelling pilot results of many app studies have not translated into clinical practice [35,39]. Standardized methods to collect, analyze, and report digital mood/behavioral data as well as clear frameworks regarding privacy and security of data are still not available [40,41], making the implementation of feasibility and validation studies an important step of this process.

### Objectives of This Study

In order to tackle some of these questions as well as to contribute to the evidence on digital phenotyping in bipolar disorder, we developed the *Toi Même* mHealth platform, which comprises the *Toi Même* smartphone app to self-monitor subjective and objective parameters of bipolar illness activity. The name of this project was inspired by the foreword engraved on the frontispiece of the Temple of Delphi—“Know thyself” (ie, *Connais toi toi-même,* in French). This expression was adopted by the philosopher Socrates who uttered it in his dialogues with his mentor Platon: “Know thyself as the dweller of the mind, senses, and the body” [42]. Our research protocol was carried out in real-world clinical settings with patients with bipolar disorder and we aimed to investigate the correlations between the clinically rated mood symptoms and mood/behavioral data that were automatically collected using the *Toi Même* app in patients with bipolar disorder presenting with different mood episodes. This study also aimed to assess the feasibility of this app for self-monitoring subjective and objective mood/behavior parameters in these patients.

## Methods

### Study Design

This is an applied research study on digital technology, software development, and analysis of app feasibility through an open-label, prospective, and multicenter clinical trial. The 3 investigation centers involved in this study are The Therapeutic Center for Bipolar Disorder (Centre Thérapeutique de Jour-Troubles Bipolaires, CTPJ-TB), Clinique Bellevue, Meudon, France; Clinique du Château de Garches, Garches, France; and the Service Hospitalo-Universitaire GHU Sainte-Anne, Paris, France.

### Study Population

The enrollment of the patients started in April 2018 at the CTPJ-TB investigation center. The inclusion criteria for this study were male or female adult patients with bipolar disorder (age, 18 years and above), diagnosed with bipolar disorder type I or II (Diagnostic and Statistical Manual of Mental Disorders, 5th edition [DSM-5] criteria), owning an iPhone (iPhone operating system 9.0 or higher) with wireless internet access, and with a sufficient level of understanding to follow the research protocol. All patients were assessed using a standardized semistructured clinical interview and self-reported questionnaires conducted by a trained psychiatrist. The exclusion criteria were the current DSM-5 diagnosis of schizophrenia, psychotic disorders, dementia, or mental retardation, and patients presenting with suicidal behavior/ideation. At the CTPJ-TB, an independent psychiatrist researcher (AAD) asked the patients if they would like to participate in the study and explained the aims of the study. If the patient agreed to participate, an informed consent form was handed out and signed by both the participant and the psychiatrist investigator (EM) who was blinded to the smartphone data. No rewards or incentives were offered to the patients for participating in the study, and all interviews and follow-up assessments were carried out by the same investigator. The Research Ethics Committee (Comité de Protection des Personnes, Ile-de-France VII) approved the study protocol ID-RCB: 2017-A02450-53, which is also registered at www.clinicaltrials.gov (Identifier: NCT03508427).

### Clinical Assessments

Data on sociodemographic and clinical assessments were performed at baseline and after 2 weeks, 1 month, 2 months, and 3 months. These are the time points at which clinical evaluation is routinely performed at the CTPJ-TB. Hence, the study assessments did not add any extra burden on the participants or extra contact time with the staff.

The severity of the depressive and manic symptoms was evaluated using the Montgomery-Åsberg Depression Rating Scale (MADRS) [43], a 10-item questionnaire, ranging from 0 to 60, with higher scores indicating more severe depression, and the Young Mania Rating Scale (YMRS) [44], a 11-item questionnaire, ranging from 0 to 60, with higher scores indicating severity of (hypo)manic symptoms. Overall functioning was assessed using the Functioning Assessment Short Test (FAST), which encompasses 24 items to evaluate 6 functional domains: autonomy, occupational functioning, financial issues, interpersonal relationships, leisure time, and cognitive functioning. FAST scores range from 0 to 72, and higher scores indicate poorer functioning and greater disability [8].

In addition to the clinical evaluations, participants completed self-report evaluations of mood symptoms, behavior, and sleep patterns by using validated instruments. Depressive and manic symptoms were self-assessed using the 16-item Quick Inventory of Depressive Symptomatology Self-Report (QIDS-SR) [45] and the Altman Self-Rating Mania, a 5-item scale [46], respectively. Sleep patterns were assessed using the Pittsburgh Sleep Quality Index [47], which differentiates “poor” from “good” sleep by assessing 7 sleep domains, that is, quality, latency, duration, habitual sleep efficiency, sleep disturbances, use of sleep medication, and daytime dysfunction. A global score of 5 or greater indicates sleep disturbances. Levels of activation were measured using the Multidimensional Assessment of Thymic States, a 20-item self-report instrument that assesses the levels of activation uncoupled from mood during the preceding week [48]. It quantitatively evaluates 5 dimensions, namely, emotional reactivity, sensory perception, psychomotor activity, motivation, and cognition, each of which can vary from hypoactivation to hyperactivation [7]. At the end of each evaluation, the investigators asked patients to give feedback regarding the overall satisfaction, ease of use, and perceived helpfulness of the *Toi Même* app (ranging from 1=not at all to 5=extremely).

The clinical data were collected using the Research Electronic Data Capture (REDCap) software [49], which is a widely used data collection platform. REDCap follows the international rules for validation, qualification, and security gold standards, which are also aligned with the Pasteur Institute’s privacy/security rules for data collection.

### *Toi Même* App

Rather than only accessing the categories of mood symptoms, the *Toi Même* app expands on the existing digital self-assessments for bipolar disorder by integrating ecological momentary assessments of the fundamental dimensions of behaviors (eg, motor activity, cognition, affective response) in the same tool through 2 gamified tests, which were created and adapted by the first author (AAD) in order to assess cognition speed (QU*i*CKBRAIN) and affective responses (PLAY*i*MOTIONS) in real-life contexts. These games also passively collect data regarding user’s response time (seconds) and the number of hits/errors in each game trial, continuously measure the daily motor activities (eg, number of steps, distance) using the smartphone’s motion sensors, and perform a comprehensive weekly assessment, including validated self-rating scales [45,46,48]. Moreover, this app is special because *Toi Même* is the first app developed in France without commercial purposes, and it has been developed specifically for monitoring bipolar illness activity.

The principles, content, and the specifications of the *Toi Même* app were conceived by AAD. After a year of collaborative work among psychiatrists, neuroscientists, software engineers, and designers, the *Toi Même* app 1.0 version used in this study was available in French and was free of charge for the study participants. The information technology department of Pasteur Institute provided all the technical support for coding the app and the platform’s back office. The *Toi Même* app functionalities are intended to be minimally invasive to the users’ daily routine and normal smartphone usage. After the patients were included in the study, patients received an email with a link to install the *Toi Même* app in their own smartphones. Once the app was installed, users could configure it to receive a notification to perform their daily/weekly assessments. The system automatically sends a reminder notification once if the users have not completed their assessments. The app allows collecting mood/behavior information in both active (ie, need the user’s action) and passive ways (ie, without the user’s action), which is captured using the smartphone sensors. Once a day, the app prompts the user to score a short graphic 8-item test, including the assessment of subjective levels of mood, energy, emotion, irritability, anxiety, motor activity, speech, and thought speed on a scale from –3 to +3. Sleep time (hours), daily events (yes/no), and medication intake (yes/no) are also assessed daily. The app also randomly delivers to the users each one of the games twice a week.

**Figure 1:**
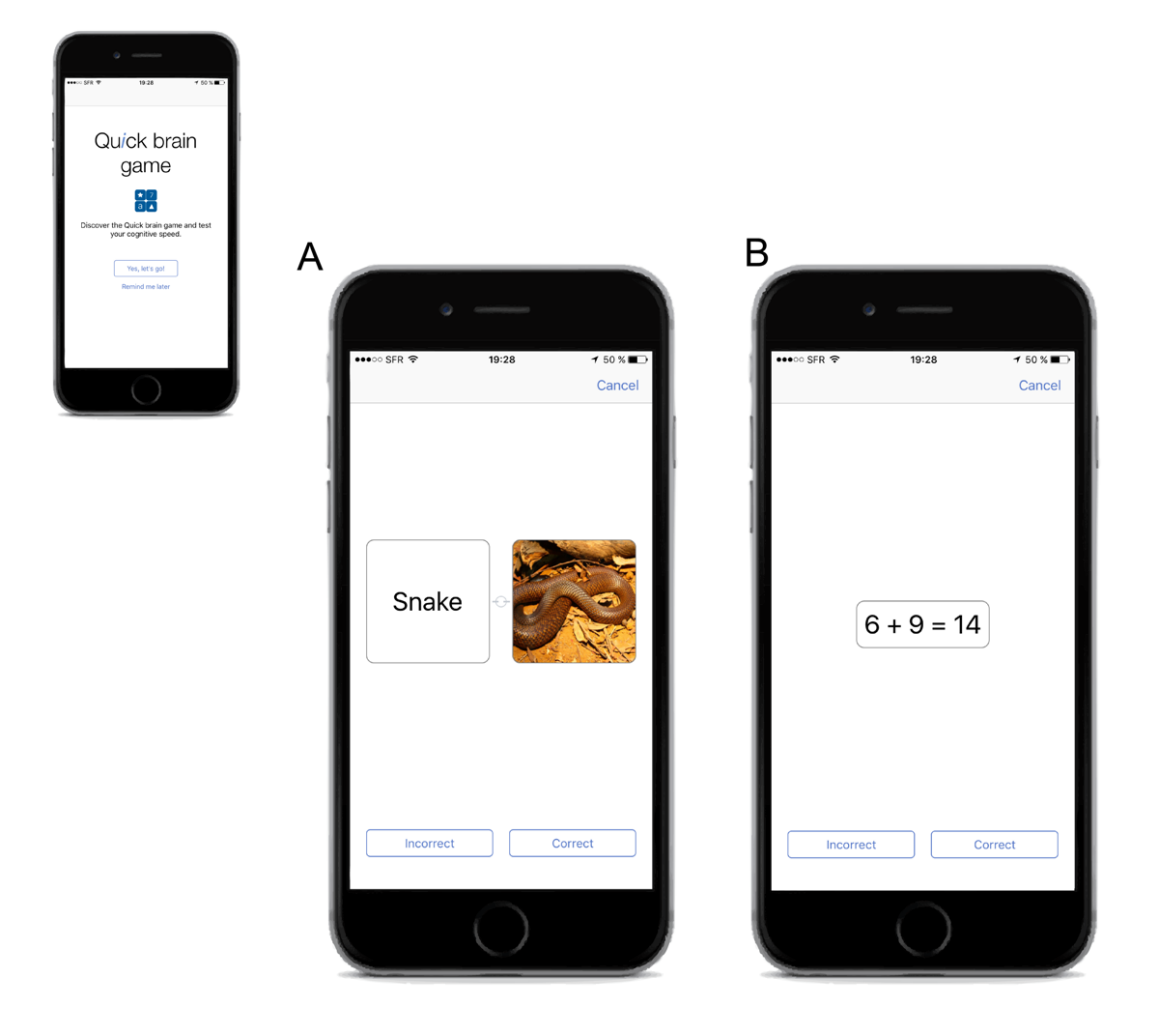
Example of a cognitive speed assessment by the QU*i*CKBRAIN task.

The game QU*i*CKBRAIN randomly offers a series of 7 trials, each one containing a pair of word image or a simple calculation. The user’s action is to analyze and answer if the word matches (or not) with the image (Figure 1A) or in case a calculation is presented, the user must answer if the result of the calculation is correct (or not) (Figure 1B). Correct and incorrect buttons are present in each trial. The QU*i*CKBRAIN task contains about 1000 different trials.

**Figure 2:**
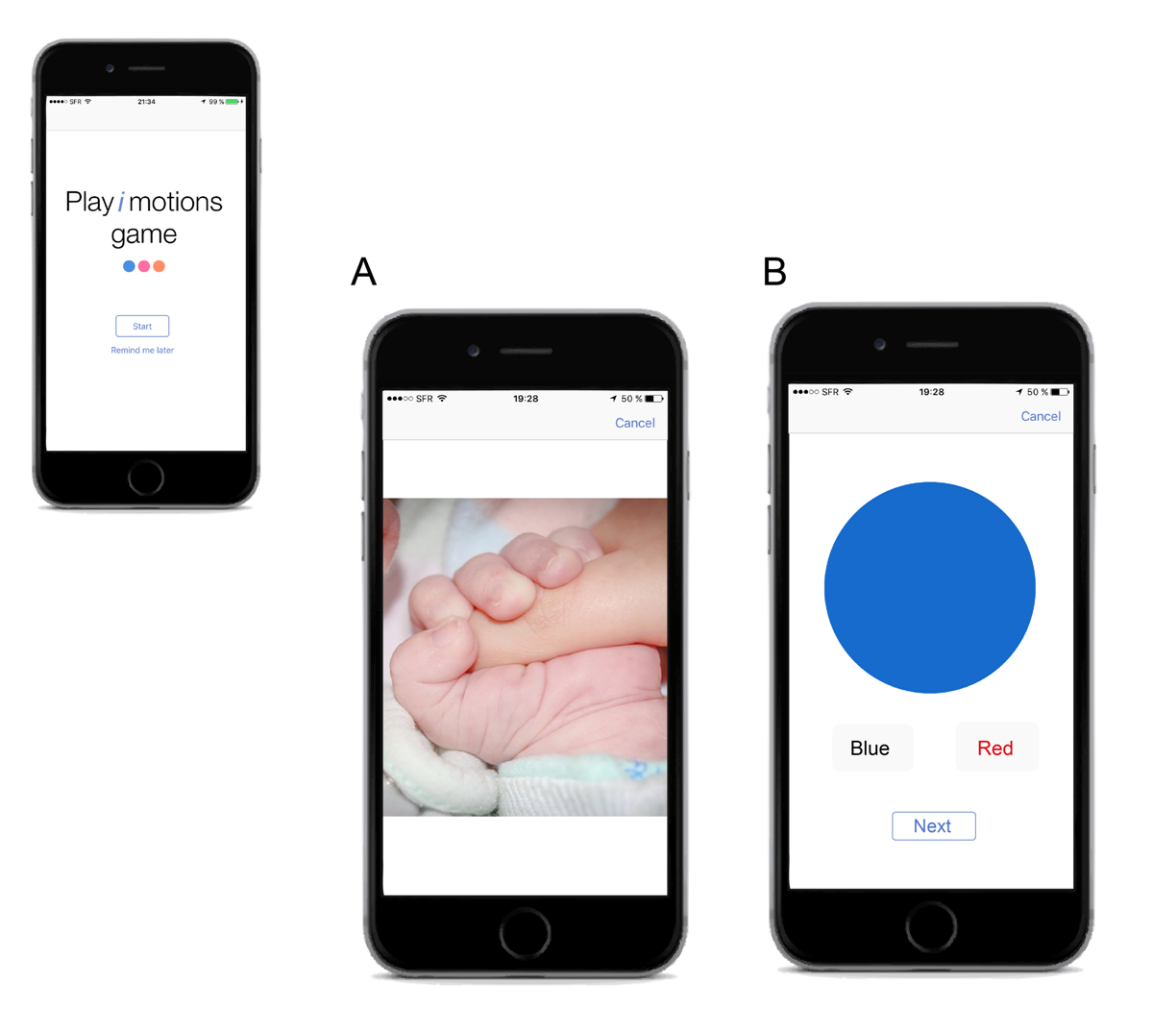
Example of an affective response assessment by the PLAY*i*MOTIONS task.

The game PLAY*i*MOTIONS is intended to assess the user’s affective response to the images. This test comprises about 1000 color images depicting a broad spectrum of themes, including humans, animals, objects, and scenes along with normative ratings on the affective/valence dimension (ie, the degree of positive, neutral, or negative affective response that the image evokes). In each game trial, an image appears on the smartphone screen for approximately 5 seconds (Figure 2A). Thereafter, the image fades away, after which a colored circle and 2 buttons appear: one button contains the name of the color of the circle written in the same color (or in a different color) as that of the circle and another button contains the name of a color different from that of the color of the circle. In this task, the user’s action is to analyze the 2 options offered and choose the one that corresponds with the color of the circle displayed on the screen (Figure 2B).

**Figure 3:**
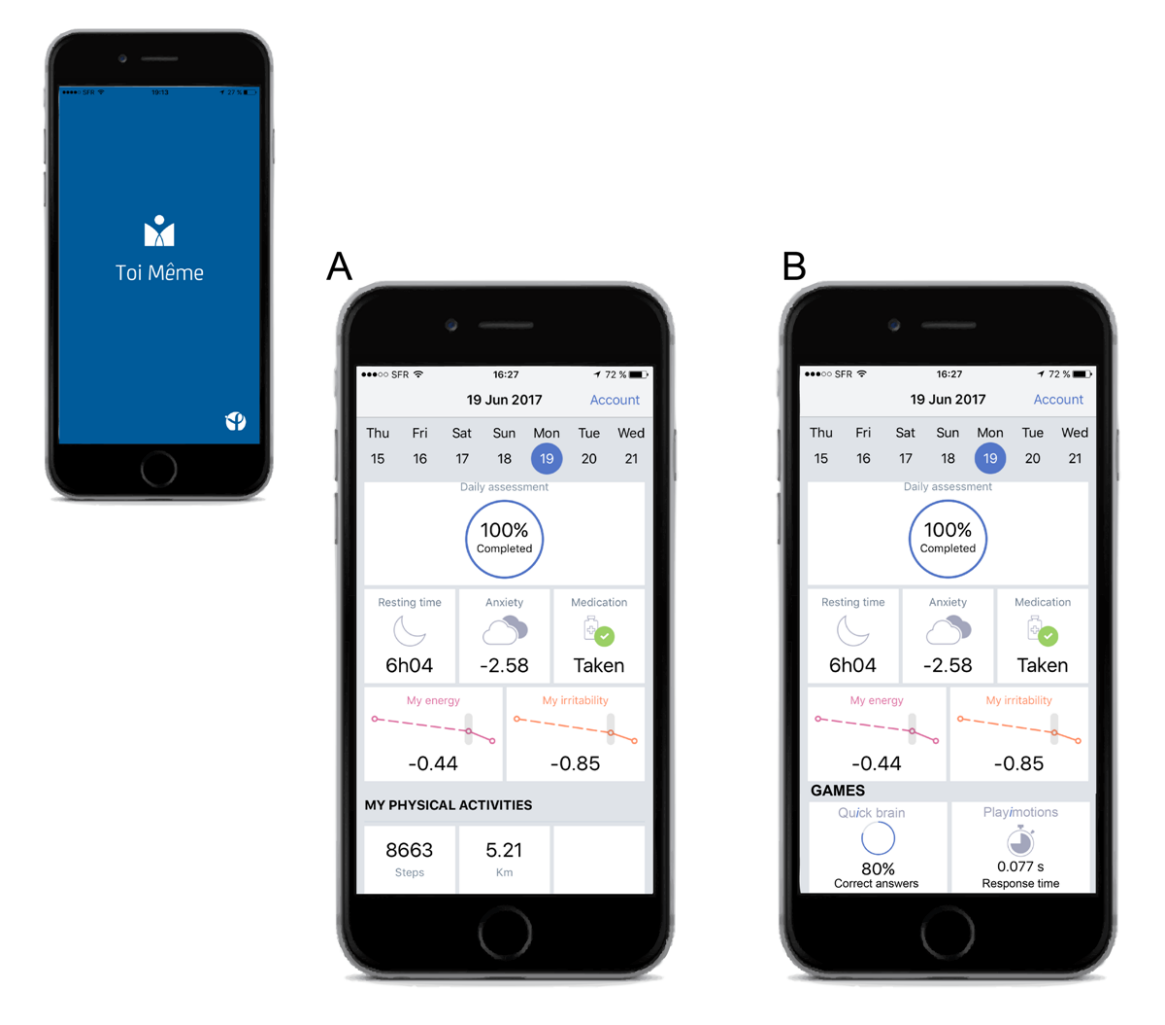
Example of the *Toi Même* app dashboard.

At the end of the daily assessment, the *Toi Même* app provides an intuitive dashboard reflecting some of the daily scores, the user’s mood/behavioral patterns during the last 7 days and, by data push, during the last 30 days (Figure 3). No diagnostic or therapeutic feedbacks are provided. All data recorded by the app are encrypted, synchronized, and stored in a deidentified format within the *Toi Même* server.

### Outcome Measures

Outcome measures are assessed at the baseline visit and after 2 weeks, 1 month, 2 months, and 3 months [50]. Briefly, primary outcome measures focus on changes in the severity of the depressive and manic symptoms assessed using the MADRS and YMRS. Secondary outcomes include changes in the self-rated mood/behavioral symptoms and medication adherence assessed using the self-report instruments QIDS-SR, Altman Self-Rating Mania, Multidimensional Assessment of Thymic States, and the Medication Adherence Rating Scale [51]. Functioning will be assessed using the FAST scale. Feasibility measures include the proportion of eligible participants who have consented to participate in the study, the frequency of self-assessments, and the proportion of participants who continued using *Toi Même* app during the study period. The participant’s physical activity will be inferred using the number of steps and distance automatically captured by the smartphone motion sensors.

### Sample Size

Considering that the focus of this pilot study is to have patients with bipolar disorder in different mood states (depression, euthymia, and hypomania), a conservative approach for calculating the sample size was adopted (ie, concordant pairs) to ensure more qualitative feedback [52]. The overall mood/behavior states assessed using the *Toi Même* app encompasses 8 dimensions of behavior (ie, mood, energy, emotion, irritability, anxiety, activity, speech, and thought speed). Each of these dimensions is rated using a score ranging from –3 to +3, yielding a total score of 24. Accordingly, a concordant pair between the app measurements and the clinical assessments of mood symptoms was defined as follows: (1) depression, a score of <–8 in the app assessments and scores of >15 for MADRS and <8 for YMRS; (2) euthymia, a score between –8 and +8 in the app assessments and scores of <15 for MADRS and <8 for YMRS; (3) (hypo)mania, a score of >+8 in the app and scores of <15 for MADRS and >8 for YMRS. Assuming that the expected proportion of the concordant pairs is 98%, with an estimated precision of 5% and a two-sided 95% CI, 31 patients will be included in each of the 3 groups to fully accomplish the study goals (N=93).

### Statistical Analysis

Descriptive analyses will be conducted to characterize the sociodemographic and clinical characteristics of the initial sample as well as the retention at the end of the study. Receiver operating characteristics analysis will be applied to calculate the concordance ratio of the pairs (ie, the area under the curve) [53]. Given that patients have started using the app from the baseline visit, the clinical data collected at this time point will not be included in the concordant pairs’ analysis. Compliance will be calculated as the proportion of the enrolled patients completing the first 15 and then 30 and 90 consecutive days of ratings. The app completion rate would be completing at least 70% of the momentary assessments during the time a participant has continued in the study. The average ratings of the perceived helpfulness, ease of use, and overall satisfaction would be at least 3 on the 1 to 5 rating scores. Cluster analysis will be performed using principal component analysis (PCA) to identify groupings of the daily *Toi Même* mood/behavior questions. The PCA results will be then used to obtain a single summary statistic (*TM*P1) that will account for the maximum amount of variability in the 8 daily *Toi Même* mood/behavior responses across all the subjects during the study period. The summary statistic produced from the PCA-*TM*P1 will be used as a response to test for differences between groups in daily ratings and for comparison between this app score and the clinical rating scores. To test for differences between groups over time, linear mixed regression models will be used with autoregressive covariance adjustments for repeated measurements of continuous outcomes and logistic generalized estimating equations will be used for binary outcomes. In both linear and logistic generalized estimating equation models, adjustments for sex, age, day of the week, and elapsed time in the study will be included as other covariates in the models. The regularity in the daily ratings for each subject will be estimated using the root mean squared successive differences (a measure of the variability of the changes in scores over time), the Teager-Kaiser Energy Operator, which combines amplitude and frequency, and the information entropy, which measures regularity of amplitude [37]. The within-subject sample variance will be also used as a measure of regularity for each subject’s response and will estimate the distribution of the amplitudes recorded over time. Data analysis will employ the intention-to-treat principle by including all participants in the analyses, which will be conducted using the R programming language (R Core Team).

## Results

The recruitment of patients started in April 2018 and the completion of the study is estimated to be in December 2021. As of April 2019, 25 participants were enrolled in the study. The first results are expected to be submitted for publication in 2020. The association between performance on both the QU*i*CKBRAIN and PLAY*i*MOTIONS and subjective cognitive/emotional functioning difficulties will be evaluated in a subsequent specific study. In the future, the transfer of technology from the academic environment to the clinics may be a strategy to invigorate efforts, making available the *Toi Même* mHealth platform for individuals at risk of developing bipolar disorder or for those experiencing bipolar disorder. This project has been funded by the Perception and Memory Unit of the Pasteur Institute (Paris) and it has received the final ethical/research approvals in April 2018 (ID-RCB: 2017-A02450-53).

## Discussion

To our knowledge, this is the first study in France that has investigated correlations between clinically rated mood symptoms and mood/behavioral data automatically collected using a smartphone app specifically developed for patients with bipolar disorder through a registered clinical trial carried out in real-world bipolar disorder treatment clinics. This multidimensional digital phenotyping approach may help to improve our understanding of bipolar illness activity and potentially contribute to early detection of subtle mood/behavior changes and to objective assessment of the response to treatment and its impact on mood, cognition, emotion, and motivation in patients with bipolar disorder. In addition, the use of digital technology in bipolar disorder treatment clinics may encourage patients with bipolar disorder to monitor their health as well as reinforce the patient-clinician therapeutic alliance.

Although the *Toi Même* project is not the first to implement a smartphone-based self-monitoring tool for patients with bipolar disorder, this app was designed as an independent self-assessment tool targeting mainly the fundamental dimensions of behavior (eg, activity, energy, cognition speed, affective responses) rather than as an augmenting intervention measuring essentially mood symptoms. The *Toi Même* clinical trial has carefully followed the ethical and legal issues around providing and obtaining adequate informed content from participants, personal privacy, and safety of data collection/storage. The same clinical psychiatrist who was blinded to smartphone data collected during the study assessed the patients by using standardized rating scales. The *Toi Même* app automatically integrates behavior data collection (eg, physical activity, user’s response time) and 2 gamified modules targeting cognitive speed (QU*i*CKBRAIN) and affective response to images (PLAY*i*MOTIONS).

Smartphones are able to automatically collect a large amount of complex and diverse data that are quickly generated and could represent a paradigm shift for accessing mood/behavior in patients with bipolar disorder, thereby providing opportunities for investigation and hypothesis generation [54,55] as well as for enhancing the precision of clinical algorithms, which today are essentially based on patients’ subjective self-report and clinicians’ observations. Promising progress in big data analytics (eg, machine learning techniques) as well as intensive multidisciplinary collaboration, including clinicians, neuroscientists, engineers, and data scientists, could further help to solve this complex puzzle, extracting meaningful indicators of bipolar disorder onset, course, and treatment response [56,57]. Future studies investigating the use of combined automatically generated smartphone data with biological and clinical measures may lead to the discovery and validation of digital markers of risk, staging, treatment response, and prognosis in patients with bipolar disorder [58].

Owing to resource constraints, this research protocol has included a *Toi Même* app version using only the iPhone operating system; hence, Google’s Android users as well those using smartphones with other types of operating systems were excluded. This could represent a sampling bias since smartphone ownership could be related to income status, education levels, and gender [14]. Three months of follow-up may be a short time frame to evaluate the feasibility of a digital self-monitoring tool for patients with bipolar disorder [39]. However, to date, there is no consensus on how long these types of interventions should be offered. Moreover, there is a multitude of smartphones in the market in which are embedded diverse sensors as well as different operating systems, which have different permissions rules to capture passive information (eg, the iPhone operating system has more restrictions than the Android operating system). To overcome this issue, research groups using digital phenotyping in psychiatry should keep trying to communicate with information technology companies regarding the development of clear and ethical frameworks for obtaining access to more homogeneous automatically generated smartphone data in order to evaluate their validity, sensitivity, and specificity for monitoring bipolar illness activity.

In summary, the *Toi Même* study is an example of the use of digital phenotyping in bipolar disorder clinical practice that can provide fine-grained mood and behavioral patterns of bipolar illness activity in real time. This clinical neuroscience–based research may add to the evidence of exploring other alternatives toward a more integrated approach, including digital phenotyping, in the management of bipolar disorder to develop a clinically meaningful framework for investigating, diagnosing, and treating individuals at risk of developing bipolar disorder or currently experiencing bipolar disorder.

## Abbreviations

AMoSS: Automated Monitoring of Symptoms Severity
CTPJ-TB: Centre Thérapeutique de Jour-Troubles Bipolaires
DSM-5: Diagnostic and Statistical Manual of Mental Disorders, 5th edition
FAST: Functioning Assessment Short Test
MADRS: Montgomery-Åsberg Depression Rating Scale
mHealth: mobile health
MONARCA: MONitoring, treAtment and pRediCtion of bipolAr Disorder Episodes
PCA: principal component analysis
QIDS-SR: Quick Inventory of Depressive Symptomatology Self-Report
REDCap: Research Electronic Data Capture
YMRS: Young Mania Rating Scale
